# Platelet Changes in Pregnancies with Severe Early Fetal Intrauterine Growth Restriction

**DOI:** 10.3390/medicina57121355

**Published:** 2021-12-12

**Authors:** Anca Marina Ciobanu, Anca Maria Panaitescu, Nicolae Gica, Ana Maria Scutelnicu, Alexandra Bouariu, Mihaela Roxana Popescu

**Affiliations:** 1 Department of Obstetrics and Gynecology, Carol Davila University of Medicine and Pharmacy, 020021 Bucharest, Romania; anca.panaitescu@umfcd.ro (A.M.P.); gica.nicolae@umfcd.ro (N.G.); roxana.popescu@umfcd.ro (M.R.P.); 2 Filantropia Clinical Hospital, 011171 Bucharest, Romania; ana.scutelnicu@yahoo.com (A.M.S.); Alexandra.bouariu@yahoo.com (A.B.); 3 Cardiology Department, Elias University Hospital, 011461 Bucharest, Romania

**Keywords:** platelet changes, mean platelet volume, fetal growth restriction, placental insufficiency, preeclampsia

## Abstract

*Background and Objectives:* In this study, we investigated the changes of platelet count and other platelet indices, such as mean platelet volume (MPV), in cases with severe early intrauterine fetal growth restriction (IUGR). *Materials and Methods*: We retrospectively analyzed all pregnancies diagnosed with severe early onset IUGR, that were followed up in our hospital between 2010 and 2015 (before implementation of screening and prophylaxis with aspirin). Pregnancies which resulted in birth of a newborn with a birthweight less than 5th percentile for gestational age, that required delivery for fetal or maternal indication before 32 weeks, were selected for the IUGR group. The IUGR cases were divided into two groups according to preeclampsia (PE) association. All cases with a complete blood count (CBC) performed within 7 days prior to delivery were included in the study, as the IUGR group. The control group included normal singleton pregnancies, delivered at term, with birthweight above 10th centile and a CBC taken at 30–32 weeks. *Results*: There was a significant difference in platelet count and MPV values between the IUGR group and control. Cases with IUGR presented lower platelet count and higher MPV values; there was no significant difference of these parameters when PE was associated with IUGR. *Conclusions***:** Our results suggest that in cases of severe early IUGR, even in the absence of clinically diagnosed PE, there may be maternal endothelial damage and platelet consumption in the systemic and uteroplacental circulation. Platelet count and MPV values are simple and widely available laboratory tests that might be used as indicator of placental insufficiency; however, prospective data are required to establish the mechanistic link and to which extent these parameters are good predictors of severity or adverse perinatal outcomes.

## 1. Introduction

Intrauterine growth restriction (IUGR) is a major cause of perinatal mortality and morbidity and complicates 3–7% of pregnancies. Unfortunately, there are no effective screening strategies or proven preventive and therapeutic methods. This may be due to the fact that the pathophysiology of IUGR is complex and not fully understood. The most common cause of IUGR, involved in more than 80% of cases is related to uteroplacental insufficiency, the same complex mechanism that leads to preeclampsia. Inadequate cytotrophoblast invasion causes endothelial cells damage and consequently increased activation of platelets at the site of vascular injury. The increased consumption of platelets in the uteroplacental and systemic circulation stimulates the bone marrow to produce more immature platelets, which are characterized by a larger volume and a higher tendency to aggregate. In a simple blood, test these changes, produced at the microvascular level, are translated into reduced platelet count below 150.000/uL and increased mean platelet volume (MPV), which is a measurement of the average platelet size and an indicator of platelet production rate. So far, it has been shown that the MPV is a marker of inflammation and especially inflammation at the microvascular site; therefore, it is related to vascular diseases such as acute myocardial infarction, atherosclerosis, diabetes, coagulopathy and hypertension [1,2].

In normal pregnancy there is a physiological increase in platelet aggregation and subsequently a decrease in the number of platelets which becomes evident towards late gestation, known as gestational thrombocytopenia. Physiologically, MPV decreases from the 20th to the 31st week of gestation and increases from the 38th week [3].

Although apparently IUGR and preeclampsia share the same pathophysiological mechanism, namely impaired trophoblast invasion, the pattern of changes in platelet size, count and function may be different and disease specific.

Norris et al. analyzed platelet count and platelet aggregation between three groups: pregnancies with IUGR and hypertensive disorders, those with normotensive IUGR and the control group. The results showed that in the group with IUGR and high blood pressure, the platelet count was reduced by 30% compared with normal pregnancy, while the aggregation was increased by 50% prior to delivery. In the normotensive IUGR group, platelet count and platelet function were similar to those found in the control group. The results support the idea that, in the normotensive group, the platelet activation is confined to the uteroplacental circulation, while in the hypertensive IUGR group the mechanism is systemic and the bone marrow production cannot compensate the platelet destruction resulting in reduced platelet count [4].

Additionally, in a recent study there was no difference in platelet indices, mainly MPV values, between IUGR and normal pregnancies. Regarding the MPV and perinatal complications the only relation was between higher maternal MPV values and neonatal respiratory distress syndrome or neonatal intensive unit admission [5].

The aim of this retrospective study was to compare changes in platelet count and platelet indices—mean platelet volume, platelet distribution width (PDW) and plateletcrit in women with severe early onset IUGR that required delivery before 32 weeks and uncomplicated singleton pregnancies.

## 2. Materials and Methods

We retrospectively analyzed all women that were followed up at Filantropia Hospital, a tertiary obstetric care center in Bucharest, between 2010 and 2015, and selected the cases with early onset severe IUGR, defined as estimated fetal weight below the 5th percentile for gestational age, that required delivery before 32 weeks of gestation for either abnormal Doppler changes, according to the TRUFFLE study [6] (abnormal ductus venosus or changes on computerized cardiotocography monitoring), or for maternal indication. All cases with these criteria and with a complete blood count (CBC) performed within 7 days prior to delivery were included in the study as IUGR group. All cases in the study group had severe early IUGR and abnormal Doppler measurement in the uterine arteries (high pulsatility index) and umbilical arteries (absent or reversed diastolic flow). Gestational age was calculated either from the first day of last menstruation or first trimester crown–rump length measurements.

Further, among early onset IUGR cases, we identified the cases complicated by preeclampsia, defined according to the American College of Obstetricians and Gynecologists (ACOG) criteria [7], as blood pressure of 140/90 mmHg or higher and proteinuria >= 300 mg/24 h or >= 2+ on dipstick, or one sign of organ dysfunction (serum creatinine is >97 µmol/L, transaminases are more than twice the upper limit of normal (≥65 IU/L for our laboratory) and platelet count is <100,000/µL, there is pulmonary edema or new-onset headache or visual disturbances). The pregnancies with congenital malformations or chromosomal abnormalities were excluded. Only deliveries with complete relevant data were included in the analysis. Anonymized patient data were used, and no individual informed consent was required. The study was undertaken in accordance with the hospital’s Ethical Committee regulations.

There were 27 cases of severe early IUGR identified that were further divided in 2 subgroups, based on associated preeclampsia, 19 cases associated with PE (IUGR–PE group) and 8 normotensive, non-preeclamptic cases (IUGR group). The control group included 35 healthy pregnant women at a median gestational age of 31 weeks with an estimated fetal weight >10th percentile delivered at term. Doppler studies of the uterine and umbilical arteries were normal during pregnancy in the control group. The blood for CBC was collected at 30–32 weeks. These were uncomplicated singleton pregnancies and patients with chronic disorders, such as chronic hypertension, diabetes mellitus, heart disease, renal or hepatic dysfunction were excluded.

We deliberately included only cases of severe early IUGR diagnosed between 2010 and 2015; this was because, at that time, screening for preeclampsia and fetal growth restriction (according to the Fetal Medicine Foundation algorithm) and subsequent prevention with low dose aspirin for patients identified at high risk [8] were not implemented into routine clinical practice. Including cases before aspirin use, we aimed to avoid any potential interference with the incidence of preeclampsia or IUGR and with the platelet changes.

Relevant demographic data, such as maternal age, parity, gestational age at delivery and birth weight were retrieved from the hospital records for analysis (Table 1).

The venous blood samples were collected in EDTA-containing tubes and the CBCs for all patients included in the study were performed in our institution using the same automated analyzer Cel-Dyn Ruby. Between the three groups (IUGR–PE, IUGR and control) we compared platelet count and platelet indices, firstly MPV, but also PDW and plateletcrit. In cases where platelet count was less than 100,000/µL, we performed manual count (peripheral blood smear) to confirm the results.

Statistical analyses were carried out using Microsoft Excel. Continuous variables were assessed for normality using the Shapiro–Wilk test. Data were expressed as median (interquartile range (IQR)) for continuous variables and *n* (%) for categorical variables. *T*-test and χ^2^-square test were used for normally distributed continuous and categorical variables, and Mann–Whitney U-test to compare variables with non-normal distribution. The statistical significance was considered for a 0.05 significance level (*p* < 0.05).

## 3. Results

**Figure 1:**
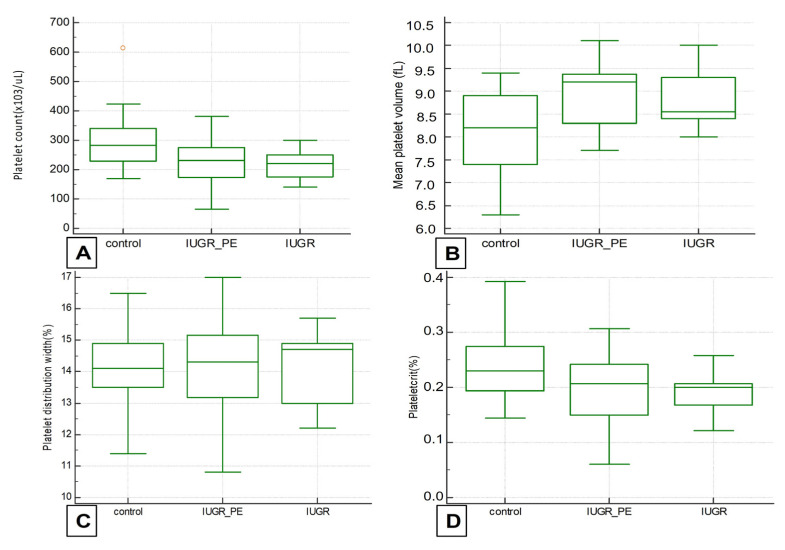
Box and whisker plots of platelet count (**A**), mean platelet volume (MPV) (**B**), plateletcrit (**C**) and platelet distribution width (PDW) (**D**) in normal control pregnancies, IUGR–PE group and IUGR alone.

In the IUGR group, the mean gestational age at delivery was 29 weeks (IQR 26–32). Despite severe prematurity, there were 25 live births, 1 intrauterine death and 1 perinatal death. The mean birth weight was 995 g (IQR 650–1500). The two IUGR groups were compared with each other and with the control group in terms of platelet count and platelets indices MPV, PDW and plateletcrit (Figure 1).

The results regarding platelet count showed a significant difference when IUGR groups were compared with control but not with each other. Interestingly, the median value of platelet count in the normotensive IUGR group was lower than in the IUGR cases associated with preeclampsia, but without a statistical significance. The lowest platelet value was observed in the IUGR-PE group in one case with severe PE which developed HELLP syndrome (66.000/uL). The same case presented the highest MPV value (10.1 fL). This finding may prove significant in a larger group.

Mean platelet volumes were significantly higher in both IUGR groups when compared with control groups (8.9 and 8.8 fL vs. 8.0 fL, *p*-0.0007 and 0.003), but similar when the groups were compared to each other. Regarding other platelet indices, PDW and plateletcrit, there was no significant difference between groups (Table 2).

## 4. Discussion

There is a lack of data or contradictory results regarding a possible association between platelet changes and IUGR, many studies focused on the changes of platelets indices in patients with preeclampsia. Previous studies highlighted the relation between platelet count decrease, MPV increase and pregnancies complicated by preeclampsia. The largest meta-analysis on platelet changes in preeclampsia showed that MPV was significantly higher in preeclamptic than healthy pregnant women and the difference was related to severity of preeclampsia [9]. The current study demonstrated significantly lower platelet values and higher MPV in pregnancies complicated with severe early IUGR, when compared with normal controls. Our findings suggest that in severe early IUGR, even in the absence of clinically diagnosed PE, there may be maternal endothelial damage and increased platelet consumption.

MPV value of 10.5 fL or higher in the late first trimester can predict preeclampsia with a sensitivity of 66% and specificity of 63% but can be also a better indicator regarding IUGR (82% sensitivity and 60% specificity). These predictive values could be enhanced by adding PAPP-A measurement in the first trimester with a cut-off of 0.3 MoM [10].

It has been observed that changes in platelet function and especially increased MVP precede clinical onset of preeclampsia by approximately 2–6 weeks and it is a good predictor of early onset PE, even when used in the first trimester of pregnancy [11,12]. A cut-off value of 8.5 fL at 24–28 weeks of gestation can predict preeclampsia with 78% sensitivity and 86% specificity [13].

Further, several studies found that PDW increase is correlated with mean arterial pressure and could be a good predictor of hypertension severity [14]. Young et al. proposed a cut-off value of 13.5% for the prediction of PE severity [15].

Regarding the association between platelet changes and IUGR pregnancies, the results are different. Some studies support the hypothesis that, similar to PE, platelets are reduced and MPV increased in pregnancies complicated with IUGR compared with normal pregnancies. In this study, we selected cases of severe early IUGR that shares common pathophysiology with PE: inadequate trophoblastic invasion and uteroplacental insufficiency.

Doppler-measured high uterine artery resistance as a predictor marker of placental insufficiency is associated with an increased platelet volume compared with those with normal uterine artery Doppler findings, many weeks before clinical signs of preeclampsia or fetal growth restriction become clinically evident [16]. Piazze et al. analyzed 51 pregnancies with abnormal uterine artery Doppler waveforms and concluded that MPV is the only platelet marker that changed compared with pregnancies with normal Doppler. A proportion of 25 out of 51 pregnancies with abnormal flow in uterine arteries developed preeclampsia, and in those cases, MPV values were higher than in 26 pregnancies complicated with IUGR [17]. Gioia et al. proposed a cut-off value of 10 fL for MPV as a predictor for unfavorable neonatal outcome in pregnancies with abnormal uterine artery Doppler [18].

On the other hand, several studies found no difference between IUGR and normal pregnancies in terms of platelet changes and concluded that in pregnancies with growth impairment platelet activation occurs only at the placental level and bone marrow production can compensate the platelet consumption [4]. In consequence, there are no changes for platelets detectable by routine blood test.

Despite the reduced number of cases analyzed, we focused on pregnancies with severe early IUGR, a relatively rare condition, but at higher risk, requiring closer surveillance by Doppler assessment and fetal heart rate monitoring. This study is one of the few conducted in pregnancies complicated with growth restriction that showed a relationship between platelet changes and placental insufficiency as a common mechanism for preeclampsia and IUGR.

Consistent with our results, another recent study including 126 cases of fetal growth restriction at term showed that MPV values were significantly increased in pregnancies with IUGR compared with normal singleton pregnancies delivered at term. The cut-off MPV for prediction of IUGR was ≥ 10.55 fL, with a sensitivity of 59% and a specificity of 75% [19]. The fact that the most severe case had the highest MPV and the lowest platelet count supports our hypothesis, and might have statistical significance in a larger group.

There was no proved association between the severity of fetal growth restriction assessed by Doppler evaluation or perinatal complications and the degree of MPV increase [20].

It is supposed that the reason for the inconsistency between published results is the population included with various degree of severity, time and method of measurement of platelets indices. Additionally, the type of anticoagulant used can cause changes in platelet shape and consequent MPV increase [21].

In this study, we analyzed pregnancies with severe early IUGR requiring delivery before 32 weeks. A possible limitation of this study is the significant difference regarding the mean gestational age at blood collection, 29 weeks (IUGR group) vs. 31 weeks (control group). In our unit, as part of routine antenatal care, all patients benefit from a third trimester ultrasound evaluation and blood tests, including CBC, which are performed around 30–32 (mean 31) weeks and in selecting the control group, this was the closest moment to the IUGR cases. However, it is not expected that platelet indices would change significantly in a physiological pregnancy within a 2-week interval [3].

## 5. Conclusions

The results of the present study indicate that platelet count and MPV are the two platelet indices that change in IUGR pregnancies, with no statistical difference when associated or not to preeclampsia. These results suggest that in pregnant women with severe early IUGR, even in the absence of PE, there may be endothelial damage and platelet consumption in the systemic and uteroplacental circulation. Low platelet count and high MPV could be used as possible markers in prediction of pregnancies complicated with placental insufficiency.

## Figures and Tables

**Table 1:** Demographic characteristics of the IUGR cases and controls. GA—gestational age.

Characteristics	IUGR (*n* = 27)	Control (*n* = 35)	*p* Value
Age (years)	31 (28–36)	30 (27–34)	0.2
Nulliparity	22 (81%)	20 (57%)	0.001
GA at CBC (weeks)	29 (26–32)	31 (28–33)	0.003
GA at delivery (weeks)	29.9 (26.0–32.0)	39.0 (38.3–40.0)	0.001
Birth weight (g)	995 (650–1500)	3300 (3000–3600)	0.001

**Table 2:** Comparison of platelet indices between the groups. IQR—interquartile range; *—statistically significant.

Platelet Indices	Median (IQR)	*p* < 0.05
Platelet count		
Control	282 (230–338)	
IUGR–PE	230 * (176–275)	0.009
IUGR	221 * (183–249)	0.02
Mean platelet volume (fL)		
Control	8.2 (7.4–8.9)	
IUGR–PE	9.2 * (8.3–9.4)	0.0007
IUGR	8.6 * (8.5–9.0)	0.003
Platelet distribution width (%)		
Control	14.1 (13.5–14.9)	
IUGR–PE	14.3 (13.3–15.1)	0.4
IUGR	14.7 (13.1–14.9)	0.4
Plateletcrit (%)		
Control	0.23 (0.19–0.27)	
IUGR–PE	0.20 (0.15–0.24)	0.06
IUGR	0.20 (0.18–0.20)	0.05
